# Changes in proton pump inhibitor prescribing trend over the past decade and pharmacists’ effect on prescribing practice at a tertiary hospital

**DOI:** 10.1186/s12913-018-3358-5

**Published:** 2018-07-11

**Authors:** Hongli Luo, Qingze Fan, Shunlin Xiao, Kun Chen

**Affiliations:** Department of Pharmacy, the Affiliated Hospital of Southwest Medical University, No. 25 Taiping Street, Luzhou, 646000 China

**Keywords:** Proton pump inhibitor, Drug utilization, Expenditure, Pharmacist intervention, Education

## Abstract

**Background:**

Proton pump inhibitors (PPIs) remain one of the world’s most frequently prescribed medications and there is a growing number of publications on correct versus incorrect use of PPIs worldwide. The objective of this observational retrospective study was to assess changes in PPI prescribing trends over the past decade and pharmacists’ effect on optimizing PPI prescribing practice at a tertiary hospital in China.

**Methods:**

We collected the prescriptions of PPIs in our hospital from January 2007 to December 2016. Then the rate of PPI prescribing, the defined daily doses (DDDs) and expenditures were calculated and plotted to show the change in utilization of and expenditure on PPIs. Reasons behind this change and effect of pharmacists’ intervention were evaluated by investigating the rationality of PPI use through sample surveys of patients of pre-intervention (Jul.-Dec. 2015) and post-intervention (Jul.-Dec. 2016).

**Results:**

In outpatient settings, the rate of PPI prescribing remained almost constant, utilization (from 135,808 DDDs to 722,943 DDDs) and expenditure (from 1.85 million CNY to 7.96 million CNY) increased for the past ten years, dominated by oral formulations and rabeprazole. In contrast, in inpatient settings, the rate of PPI prescribing (from 20.41 to 37.21%), utilization (from 132,329 DDDs to 827,747 DDDs) and expenditure (from 3.15 million CNY to 25.29 million CNY) increased from 2007 to 2015 and then decreased, dominated by injection formulations and omeprazole. Pharmacist interventions could significantly promote the rational use of PPIs (44.00% versus 26.67%), decrease PPI use and reduce patients’ charges (*P* < 0.05).

**Conclusions:**

The utilization of and expenditure on PPIs grew due to the increase of patients and irrational use of PPI. Pharmacist interventions help to reduce PPI utilization and expenditure and enhance rationality for inpatients, but much work should be done to regulate injection and originator formulas, and improve the rationality in the future.

## Background

The rational prescription of medications is vital to ensuring the optimal use of medical resources, providing effective therapy and minimizing adverse drug reactions. In practice, a few of the most common irrational uses of medicines are excessive prescriptions of antibiotics, proton pump inhibitors (PPIs) and glucocorticoids [1–3]. Since omeprazole, the first PPI, emerged in the market in the late 1980s, PPIs have proven to be an effective therapeutic option in a variety of acid-related disorders including gastroesophageal reflux disease, peptic ulcer disease, Helicobacter pylori eradication therapy, dyspepsia and stress ulcer prophylaxis. The use of PPIs increased by 450% in the 1990s relative to earlier years [4]. There is a growing number of publications on the overuse of PPIs in clinical practice worldwide. For example, in the United States, PPIs were prescribed in 4% of outpatient visits in 2002 and 9.2% in 2009 [5]. A Spanish drug-utilization study showed that 28.65% of inpatients were prescribed a PPI on admission, 82.62% during hospitalization, and 54.75% at discharge; moreover, incorrect indications for PPIs were found in 74.47, 61.25 and 80.24% of these cases, respectively [6]. Furthermore, previous studies showed that once PPIs were started in the hospital, more than 50% of patients continued to take them 3 to 6 months after discharge, even in the absence of an indication [7–9]. The uncontrolled overutilization of PPIs resulted in the number of prescriptions for PPIs ranking 8th on the list of the top therapeutic classes by prescriptions in 2013. Meanwhile, PPIs are one of the highest-selling classes of drugs in many countries. The expenditure on PPIs is $14 billion (United States), £425 million (England), and £7 billion a year (globally), and almost £100 million in England and £2 billion worldwide are being spent unnecessarily on PPIs each year [10, 11]. More recently, research has uncovered similar phenomena regarding PPI overuse in China. A drug-utilization study showed that 35.59% of inpatients were prescribed PPIs [12]. Two other studies found that 30.95 and 76.3% of surgical patients received stress ulcer prophylaxis (SUP) with PPIs, but the majority of them (59.26 and 67.03%, respectively) had received this medication without indications and for long durations [13, 14], which was linked to economic waste. A few factors postulated to contribute to the overutilization of PPIs include the high incidence of acid-related disease, the high efficacy and relatively low toxicity profile of PPIs, the acceptable costs of treatment with both generic and originator preparations, their availability as over-the-counter drugs and consumer-oriented advertising. However, prior studies have demonstrated that PPIs are associated with pneumonia, enteric infection, osteoporosis, hypomagnesemia and drug-drug interactions [15–18]. Therefore, to prevent these complications and avoid medical waste, it is essential to implement interventions to improve the rational use of PPIs, especially for prophylactic applications.

In recent years, various measures have been taken by authorities in China to control the irrational use of medicines and to reduce patients’ medical expenditure, especially for medicines. First, “Prescription Management Measures” and “Rules for the management of the hospital’s prescription review (trial)” were established to standardize doctors’ prescription behavior and promote the rational use of drugs. Second, the catalogues of National Essential Medicine and National Medical Insurance were published by the National Health and Family Planning Commission and the Ministry of Human Resources and Social Security of the People’s Republic of China, respectively. Moreover, the National Health and Family Planning Commission dictated that pharmaceutical expenditure in public hospitals must account for less than 30% of their total incomes, while the costs of essential medicine and originator drugs must account for more than 25% or less than 20% of total pharmaceutical expenditure. Consequently, the drugs in the two catalogues and generics should be preferred. However, pharmaceutical expenditures in hospitals actually account for approximately 40 to 50% of their total income. Finally, a real-time clinical pharmacist intervention program was implemented, which could promote the rational use of antibiotics and PPIs, resulting in favorable economic outcomes [19, 20].

There are six PPIs (omeprazole, lansoprazole, pantoprazole, rabeprazole, esomeprazole and ilaprazole) available as both oral enteric-coated tablets and intravenous injections and as both generics and originators in China. These drugs are all listed in the national medical insurance catalogue, except for ilaprazole. However, only one of the six PPIs, omeprazole, is listed in the catalogue of national essential medicine. Generic PPIs are available at a lower cost than originators, and oral PPIs are similar in effectiveness to injectable PPIs at equivalent doses. Nevertheless, previous studies found considerable use of injectable and originator PPIs. In addition, PPI overuse was usually the result of inappropriate SUP in non-intensive care patients. Therefore, to promote the rational prophylactic use of PPIs and improve the prevention of stress ulcers, the “Consensus Review for SUP and Treatment” and “Prevention and Treatment of Stress Related Mucosal Disease” were published in 2015 in China [21]. PPIs were under the supervision of Health and Family Planning Commission of Sichuan Province as one of the overused groups as of February 2, 2016. Several supervision measures were formulated including a clinical pharmacist intervention. The work role of clinical pharmacists was to correct doctors’ misunderstanding, monitor medical records in real time and control the prescriptions of PPIs based on the criteria established by the hospital administration.

The primary objective of the study was to report the trends in PPI utilization and expenditure in a tertiary teaching hospital over the past decade. The secondary objective was to evaluate the impact of clinical pharmacist interventions and certain management policies for the rational use of PPIs.

## Methods

### Study design

This is a retrospective observational study of prescriptions dispensed over the past decade, between January 2007 and December 2016, in the Affiliated Hospital of Southwest Medical University. The hospital was chosen because it is the largest public hospital in the South Sichuan Province and is a 3200-bed major academic tertiary hospital with an average daily admission rate of approximately 4000 patients and > 120,000 inpatient admissions annually. In addition, the hospital is located at the intersection of the Sichuan, Yunnan, and Guizhou Provinces and Chongqing Municipality, serving a total population of 40 million people. Moreover, an appreciable number of medicines are available for prescribing, and mass data on both utilization and expenditure can be obtained. Meanwhile, as mentioned, appreciable numbers of measures related to health insurance, price and rational drug use were implemented during this period.

In total, five PPIs (Anatomical Therapeutic Chemical Classification – group A02BC) including omeprazole (originator oral, originator injectable, generic injectable), lansoprazole (generic oral, generic injectable), pantoprazole (generic oral, generic injectable), rabeprazole (originator oral, generic oral) and esomeprazole (originator oral, originator injectable) were available for analysis during the course of the study. Generic PPIs are manufactured by Chinese enterprises with local investments, but most originator PPIs are manufactured by joint ventures in China founded by global pharmaceutical companies.

The Hospital Information System and Prescription Automatic Screening System of Sichuan Medico Software Research and Development Co., Ltd., were used to collect the numbers of patients in outpatient and inpatient settings and the data related to PPI application including drug name, dosage form, specification, unit price, volume, and manufacturer from 2007 to 2016. All data collected was anonymised and could not be traced back to an individual. The rates of PPI prescribing, defined daily doses (DDDs), DDDs/1000 inhabitants per day (DDDs/TID) and expenditure were used for measuring drug utilization and expenditure, which was in line with international recommendations. DDDs are defined as “the average maintenance dose of a drug when used for its major indication in adults”. The Chinese currency Renminbi “yuan” (CNY) was used to determine expenditure for PPIs over time. They were not adjusted for inflation or deflation so as to compute actual changes during this period. 6.5 CNY equals to 1 US dollar. In order to evaluate the rationality of the change in PPI prescribing trends and analyze the further reasons behind this phenomenon, the PPI prescribing rates of different departments in our hospital were measured and sorted and the top ten departments for PPIs prescribing rates of inpatients were chosen to represent the PPIs prescribing behavior in inpatient setting. Briefly, the rationality of PPIs prescribing was evaluated by random sampling of cases in top ten departments and analyzing their PPI use. The intervention was also implemented in these departments in 2015.

### Intervention

Several interventions about the rational use of PPI including management policies and clinical pharmacist intervention introduced by the Hospital Pharmacy Administration and Therapeutics Committee were performed from March 1, 2016 to December 31, 2016. The management policies included establishing internal guideline for SUP and reward and punishment mechanism. During the intervention period, there was an appropriative clinical pharmacist in each ward. The pharmacist intervention was implemented by our previous method with some modifications [22, 23]. The work of clinical pharmacists was to correct doctors’ misunderstanding, monitor medical records and control the prescriptions of PPIs based on the criteria established by the hospital administration. In order to evaluate intervention effect, medical records were collected. All patients receiving PPI were enrolled. Patients were included if they had used PPI and had no systemic diseases. Patients were excluded because of one of the following reasons: ≤18 years, ≥80 years, died during the hospitalization, had taken PPI within two weeks prior to hospitalization regardless of whether an indication was documented in the medical chart. All medical records in top ten departments with the highest PPI prescribing rates in pre-intervention (Jul.-Dec. 2015) and post-intervention (Jul.-Dec. 2016) stage were exported and we randomly took samples of 300. Randomization was computer-generated. The rationality of PPI use in respect of indication, drug selection, dosage, administration route, solvent and duration was analyzed.

### Statistical analysis

Data were collected and subsequently analyzed by SPSS version 19.0. For comparison between the two stages, data were analyzed using chi-squared test for categorical variables, and using student’s t-test for continuous variables. A *p*-value of less than 0.05 was considered statistically significant. Charts were performed using Graphpad Prism 6.0 software.

## Results

### Rate of PPI prescribing

**Fig. 1:**
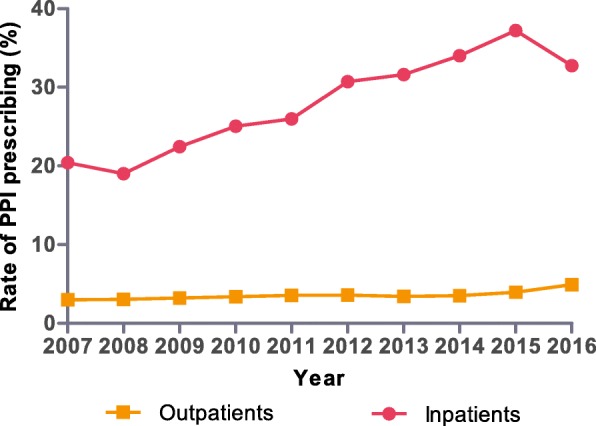
The rate of PPI prescribing for outpatients and inpatients from 2007 to 2016

The number of outpatients and inpatients had increased from 0.61 to 1.072 million, from 48,902 to 108,745 over the past ten years, and number of drugs per prescription was about three. The rate of PPI prescribing remained almost constant in the outpatient setting and increased steadily in the inpatient setting. However, the rate for inpatients decreased mildly from 2015 (37.21%) to 2016 (32.74%) (Fig. 1). The PPI prescribing rates in the top ten departments for inpatients were higher than 50% from 2013 to 2016. Interestingly, the departments with the highest PPI prescribing rates were surgical departments such as hepatobiliary or cardiothoracic surgery rather than the gastroenterology department (Table 1).

**Table 1:** The top ten departments with the highest PPI prescribing rate for inpatients from January 2013 to December 2016 (%)

Rank	2013		2014		2015		2016	
	Department	Prescribing rate	Department	Prescribing rate	Department	Prescribing rate	Department	Prescribing rate
1	Hepatobiliary surgery	80.85	Intervention	83.80	Intervention	79.68	Cardiothoracic surgery	77.44
2	Intervention	76.94	Hepatobiliary surgery	83.68	Hepatobiliary surgery	79.33	Intervention	76.48
3	Gastroenterology	75.35	Gastroenterology	76.05	Cardiothoracic surgery	76.56	Hepatobiliary surgery	74.34
4	Cardiovascular medicine	72.68	Cardiovascular medicine	72.14	Gastroenterology	70.55	Gastroenterology	73.36
5	Hematology	62.88	Cardiothoracic surgery	66.58	Cardiovascular medicine	70.34	Cardiovascular medicine	69.54
6	Infectious Disease	60.87	Urology	60.37	Neurosurgery	66.91	Neurosurgery	66.63
7	Oncology	57.72	Hematology	59.99	Oncology	60.45	Rheumatism Immunity	61.23
8	Urology	57.41	Oncology	59.72	Hematology	56.43	Respiratory	56.16
9	Gastrointestinal surgery	56.88	Gastrointestinal surgery	58.17	Respiratory	55.82	Hematology	53.80
10	Cardiothoracic surgery	56.63	Respiratory	52.95	Urology	55.72	Spinal surgery	52.12

### Utilization

**Fig. 2:**
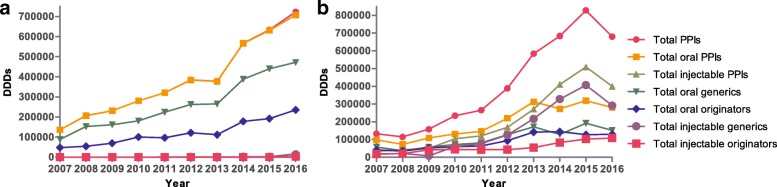
Utilization of oral and injectable PPIs for outpatients and inpatients from 2007 to 2016. DDDs, defined daily doses; **a** outpatients; **b** inpatients

**Fig. 3:**
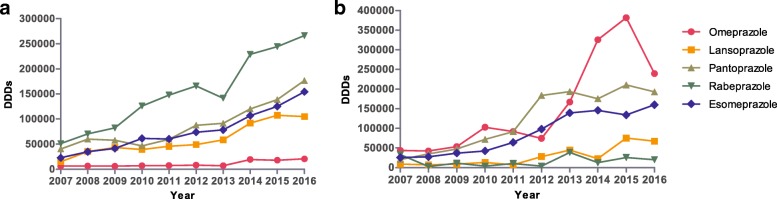
Utilization of the different PPIs (all formulations) for outpatients and inpatients from 2007 to 2016. DDDs, defined daily doses; **a** outpatients; **b** inpatients

PPI utilization increased 5.32-fold during the study period in the outpatient setting, rising from 135,808 DDDs in 2007 to 722,943 DDDs in 2016, with utilization dominated by oral PPIs. The utilization proportion of generic PPIs has remained steady at approximately 70% over the past ten years (Fig. 2a). The utilization of all forms of lansoprazole grew 6.97-fold during the course of the study compared with 6.73-fold for esomeprazole, 5.20-fold for rabeprazole, 4.38-fold for pantoprazole and 3.32-fold for omeprazole. Moreover, rabeprazole dominated PPI utilization (Fig. 3a).

**Fig. 4:**
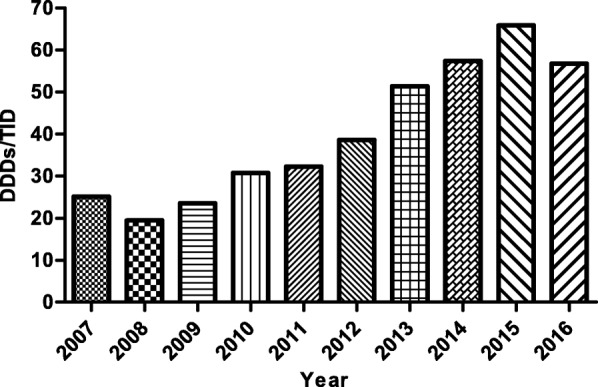
Utilization of PPIs for inpatients from 2007 to 2016 in DDDs/TID. DDDs, defined daily doses; TID, 1000 inhabitants per day

PPI utilization increased 6.26-fold over the years from 2007 to 2015 in the inpatient setting, rising from 132,329 DDDs to 827,747 DDDs (Fig. 2b). Meanwhile, DDDs/TID for inpatients grew from 25.10 in 2007 to 63.46 in 2015 (Fig. 4). The utilization of injectable PPIs increased from 26% of total PPI utilization in 2007 to 61% in 2015, but utilization was dominated by oral PPIs from 2007 to 2013. The utilization percentage of generics and originators in oral PPIs remained almost equal during the study period. Concurrent with this, there was greater growth for generic versus originator injectable PPIs, growing from 48% of total injectable PPIs in 2006 to 73% in 2016 (Fig. 2b). However, PPI utilization (679,728 DDDs and 55.17 DDDs/TID) and the percentage of injectable PPIs (59%) for inpatients decreased significantly in 2016 with the implementation of clinical pharmacist interventions (Figs. 2b and 4). Omeprazole dominated PPI utilization, with its percentage of total PPIs increasing from 33% in 2007 to 44% in 2010; however, it dramatically decreased to 19% in 2012, which was due to consideration of drug interactions. But this rate increased sharply to 46% in 2015. Fortunately, after implementing intervention the utilization of omeprazole decreased (35%) in 2016. The utilization of pantoprazole increased from 16% in 2007 to 47% in 2012, before stabilizing at 28% of total PPI utilization in 2016. The utilization of rabeprazole declined from 25 to 3% over time (Fig. 3b).

### Expenditure

**Fig. 5:**
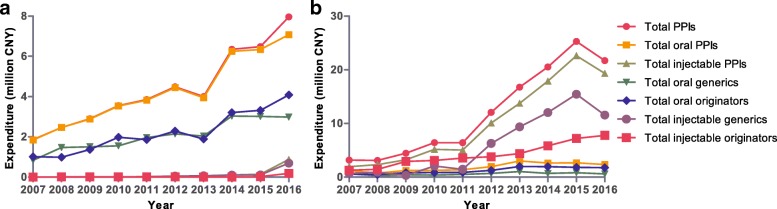
Expenditure (CNY) on oral and injectable PPIs from 2007 to 2016. **a** outpatients; **b** inpatients

**Fig. 6:**
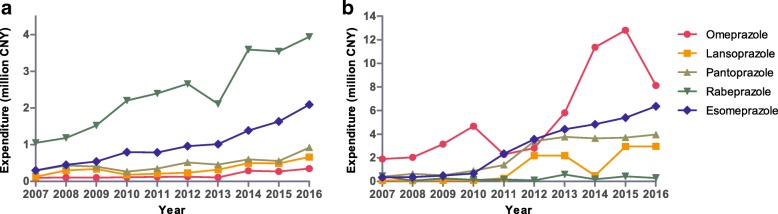
Expenditure (CNY) on the different PPIs from 2007 to 2016. **a** outpatients; **b** inpatients

The number of outpatients gradually increased between 2007 and 2012, after which it remained steady. The overall trends in Table 2 show that the total costs and average costs of all drugs and PPIs for outpatients increased from 2007 to 2016. The percentage of PPI cost grew from 2007 to 2011 but then declined during the next few years. Oral PPIs dominated PPI expenditure, and the percentage of total PPIs was nearly 100%. Oral generic PPIs and oral originators constituted approximately half of the total expenditure (Fig. 5a). Moreover, rabeprazole dominated PPI expenditure (Fig. 6a).

**Table 2:** Expenditure (CNY) for all drugs and PPIs for outpatients and inpatients from 2007 to 2016

	2007	2008	2009	2010	2011	2012	2013	2014	2015	2016
Patients (n)										
Outpatients	608,359	675,124	732,238	818,650	987,308	1,134,962	1,120,506	1,241,899	1,162,991	1,072,400
Inpatients	48,902	55,126	62,798	69,330	75,470	93,251	101,741	112,019	115,740	108,745
Total costs of all drugs (million CNY)										
Outpatients	69.53	87.33	102.88	96.68	98.78	121.68	204.01	238.84	242.52	305.85
Inpatients	124.16	146.02	173.91	226.28	271.79	390.14	476.03	458.34	617.42	610.84
PPI cost (million CNY)										
Outpatients	1.86	2.47	2.90	3.55	3.85	4.49	4.00	6.35	6.48	7.96
Inpatients	3.16	3.12	4.47	6.43	6.42	12.05	16.76	20.52	25.28	21.70
PPI cost percentage (%)										
Outpatients	2.67	2.83	2.82	3.67	3.90	3.69	1.96	2.66	2.67	2.60
Inpatients	2.54	2.14	2.57	2.84	2.36	3.09	3.52	4.48	4.10	3.55
Average cost of all drugs (CNY)^a^										
Outpatients	114.29	129.36	140.49	118.10	100.05	107.21	182.07	192.32	208.53	285.20
Inpatients	2538.93	2648.78	2769.31	3263.87	3601.34	4183.81	4678.83	4091.65	5334.54	5617.17
Average PPI cost (CNY)^b^										
Outpatients	102.88	119.57	122.68	128.52	109.84	109.90	104.86	145.72	140.64	151.45
Inpatients	316.22	298.09	317.05	370.12	327.36	420.84	521.14	538.48	587.03	609.37

^a^All patients

^b^Patients using PPIs

Similarly, the number of inpatients gradually increased between 2007 and 2012, after which it remained steady. However, the total costs and average costs of all drugs and PPIs continued to have sustained growth during the course of this study. The cost percentage of PPIs for inpatients increased from 2.54% in 2007 to 4.48% in 2014, after which it began to slightly decrease (Table 2). The growth in expenditure on injectable PPIs (11.51-fold) was greater than that on oral PPIs (2.22-fold) from 2007 to 2015, with expenditure on injectable PPIs increasing from 62% of total PPI expenditure in 2007 to 90% in 2015 (Fig. 5b). There was a primary contribution of oral originator PPIs to total oral PPI expenditure, reaching a maximum of 74% in 2014. Injectable originator PPIs dominated injectable PPI expenditure (Fig. 5b). Total PPI expenditure increased 6.87-fold during the course of the study, with expenditure dominated by omeprazole (Fig. 6b). After the intervention of clinical pharmacists, expenditures on all PPIs and injectable PPIs and the cost percentage of PPIs in total drugs in inpatient settings decreased significantly in 2016 (Table 2 and Fig. 5b).

### Evaluation of intervention effect

In order to analyze the deep reasons for PPI prescribing trends and the influence of clinical pharmacists, the appropriateness in PPI use was evaluated. General characteristics of the inpatients in two groups were shown in Table 3. The 2 groups were similar with respect to demographics and clinical characteristics, such as gender, age, body mass and length of stay (*P* > 0.05). 238 cases (79.33%) and 229 cases (76.33%) received PPI prophylaxis in pre- and post-intervention groups, respectively (*P* = 0.38). According to the established criteria for SUP and package inserts, inappropriate PPI uses in the pre- and post-intervention groups were summarized in Table 4. After intervention, the rate of irrationality (from 73.37 to 56.00%), average drug cost and average PPI cost decreased significantly (*P* < 0.05). Obvious improvements were observed in the use of no indications and inappropriate drug selection, dose, route, solvent, replacement and duration (*P* < 0.05).

**Table 3:** General characteristics of inpatients in pre- and post-intervention groups

Characteristics	Pre-intervention (*n* = 300)	Post-intervention (n = 300)	*P*
Male, n (%)	182 (60.67)	166 (55.33)	0.186
Age (yr), mean ± SD	51.23 ± 13.69	49.40 ± 14.99	0.118
Body mass (kg)	58.40 ± 9.61	56.59 ± 13.99	0.065
Length of stay (day), Mean ± SD	10.09 ± 7.17	9.91 ± 6.63	0.745
Prophylactic	238 (79.33%)	229 (76.33%)	0.380
Average hospitalization cost (CNY)	25,166.24 ± 7155.16	22,941.26 ± 6939.77	0.118
Average drug cost (CNY)	5823.88 ± 1490.91	5228.78 ± 1313.89	0.037
Average PPI cost (CNY)	657.04 ± 147.05	594.02 ± 138.76	0.032

**Table 4:** Rate of inappropriate PPI use in pre- and post-intervention groups

	Pre-intervention n (%)	Post-intervention n (%)	*P*
No indication	171 (57.00)	122 (40.67)	< 0.001
Inappropriate choice of PPI	21 (7.00)	1 (0.33)	< 0.001
Inappropriate dose	28 (9.33)	13 (4.33)	0.015
Inappropriate administration route	128 (42.67)	80 (26.67)	< 0.001
Inappropriate choice of solvent	22 (7.33)	2 (0.67)	< 0.001
Inappropriate volume of solvent	24 (8.00)	0 (0.00)	< 0.001
Unnecessary replacement of drugs	25 (8.33)	11 (3.67)	0.016
Inappropriate duration of PPI	124 (41.33)	74 (24.67)	< 0.001
Appropriate PPI use	80 (26.67)	132 (44.00)	< 0.001

## Discussion

The present retrospective survey showed that the PPI prescribing rate in the inpatient setting was 20–37%, less than that of other hospitals [5, 6]. Moreover, the vast majority of PPIs were used for SUP. In recent years, the overutilization of PPIs has become common. We also observed that approximately 80% of inpatients in the hepatobiliary surgery and intervention department received at least one PPI and most of these inpatients had no appropriate indications for the medicine [12]. Our observations were confirmed by other recently published studies [24, 25]. Fortunately, the prescribing rate, utilization of and expenditure on PPIs, PPI use with no indication significantly decreased in 2016, which was attributed to supervision measures taken by hospital administration and the implementation of clinical pharmacist interventions.

The reasons for PPI overuse were difficult to explain and likely multifactorial and may include urbanization, the expansion of the scope of medical insurance, improvements in living standards, misperceptions regarding drugs by clinicians and patients, and tense doctor-patient relationships. Some clinicians demonstrated a misunderstanding of the infrequent adverse drug events (ADEs) of PPIs and lacked attention to the serious ADEs, especially when considering the short course of PPI administration. In one survey of internal medicine physicians, less than 50% of doctors stated that they were concerned about ADEs when considering whether to prescribe PPIs [26]. Another survey revealed that 28% of attending physicians felt PPIs were “harmless” [27]. Generally, it was not cost-effective to use PPIs for SUP except in the highest-risk patients [28]. Nevertheless, because of tense doctor-patient relationships in China and to protect themselves from lawsuits, clinicians had to prescribe PPIs for SUP in low-risk patients. Thus, medical and pharmaceutical education should also be implemented with the patients to improve the doctor-patient relationship. The overuse of PPIs poses significant health risks and increases healthcare costs. However, the zero-markup drug policy has been implemented in the Sichuan Province since December 20, 2016, which has contributed to alleviating patients’ medical burden.

As shown in Table 1 and Table 3, the vast majority of PPIs were used for SUP. The recommendations related to agents for SUP were not consistent across developed high-quality clinical practice guidelines, and most guidelines recommended using PPIs or histamine-2 receptor antagonists (H_2_RAs) [29]. There was not sufficient evidence for the efficacy and safety superiority of PPIs over H_2_RAs for SUP, and since H_2_RAs were cheaper, H_2_RAs were also recommended [21], which would result in the reduction of PPI utilization. When gastrointestinal bleeding occurred and helicobacter pylori eradication therapy was applied, PPIs were the optimal selection. Overall, the utilization of and expenditure on different PPIs increased during the study period. Omeprazole, pantoprazole and esomeprazole were the three most commonly used. The utilization of and expenditure on omeprazole remained at the top position from 2007 to 2011 but then decreased sharply in 2012 and then quickly increased again starting in 2013. This interesting phenomenon was probably attributed to the discovery of drug interactions of clopidogrel and omeprazole in 2012 [30]. A systematic meta-analysis recommended avoiding omeprazole when combined with clopidogrel [31], which caused the number of prescriptions of omeprazole to decrease in some patients, whether they used clopidogrel or not. In our study, 21 cases in pre-intervention group were inappropriately prescribed the combination of omeprazole plus clopidogrel, but this combination was found in only one case in post-intervention group. However, omeprazole was listed as a national essential medicine as well as a medical insurance medicine, and its use was encouraged, which has resulted in increased utilization since 2013. A similar change was found in the Chongqing hospital during this period [32]. Generally, the new generation of PPIs such as rabeprazole and esomeprazole should be preferred in consideration of safety.

Five oral PPIs were available throughout the study period. However, the utilization of injectable PPIs was predominant. This utilization was considerably higher than the WHO guidelines for the use of injectables among developing countries [33]. According to the WHO guidelines, the oral PPIs had similar effectiveness to injectable formulations, and their prices were cheaper compared with the injections of the same dosage [34]. After analysis of the sampled cases, we found that the high utilization of and expenditure on injectable PPIs was due to the inappropriate prescribing habits in our hospital. There existed serious inappropriate administration route (42.67%) and duration (41.33%). The inappropriate administration route involves unnecessary intravenous administration when oral formulations would be more appropriate and confusing misuse of intravenous drip and intravenous injection, both of which caused the increase of injectable PPIs. This practice might be driven by the pressure of hospitals and clinicians to make money from drug dispensing, especially from the high profit of generic injections. In addition, because of the negligence of the duration under the heavy operation work and the misconception of that “the longer duration, the better efficacy”, surgeons always prolonged the duration of prophylaxis. After implementing pharmacist interventions, the utilization of and expenditure on injectable PPIs and duration were significantly reduced in 2016, but the percentage was still unsatisfactory. Thus, more measures are needed to change clinicians’ prescribing habits.

Unfortunately, our study had several limitations. First, the data in this article were collected from just one hospital. However, we believe these findings are generalizable to other hospitals in China based on the merits of our methodology as well as our hospital scale and geographical location as the largest public hospital in the South Sichuan Province. Second, the effect of H_2_RAs and gastric mucosal protective agents were not included in this study, which might have a substantial effect on the utilization of PPIs. Thirdly, our retrospective study was less convincing than a randomized controlled trial. Finally, although the proportion of rationality of PPI utilization increased during intervention phase, it (44%) was not satisfied. In a future study, we will implement a more intrinsically rigorous design to evaluate pharmacist interventions and will take further measures to reduce use of injectable PPI and shorten PPI prophylactic duration.

## Conclusion

The present study demonstrated that PPI prescribing rate and the utilization of and expenditure on PPIs increased steadily between 2007 and 2015. However, the overuse and irrationality of PPI utilization significantly decreased in 2016 under pharmacist interventions. Nevertheless, high utilization of injectable PPIs and originator PPIs and no indication continued. Consequently, regulation authorities and hospital leadership should take more potent measures to promote the rational use of PPIs, including optimizing drug procurement catalogues, supervising the implementation of ‘Prescription Management Measures’, continuous medical education for clinicians, and implementing special clinical pharmacist interventions. Once these measures are underway, they will help enhance the quality and efficiency of prescriptions. As a result, favorable clinical and economic outcomes will be attained. Finally, we hope that some measures described in this article provide examples to other provinces and municipalities throughout China as they strive to further improve the rationality and efficiency of their prescribing practices with ever greater pressure on resources.

## Abbreviations

ADEs: Adverse drug events
DDDs: Defined daily doses
DDDs/TID: DDDs/1000 inhabitants per day
H_2_RAs: Histamine-2 receptor antagonists
PPIs: Proton pump inhibitors
SUP: Stress ulcer prophylaxis

## References

[1] Webster P (2017). Antibiotic overprescribing a growing problem. CMAJ.

[2] Kazberuk M, Brzosko S, Hryszko T, Naumnik B (2016). Overuse of proton pump inhibitors and its consequences. Postepy Hig Med Dosw (Online).

[3] Atik OS (2017). Overuse and abuse of cortisone. Eklem Hastalik Cerrahisi.

[4] Guda NM, Noonan M, Kreiner MJ, Partington S, Vakil N (2004). Use of intravenous proton pump inhibitors in community practice: an explanation for the shortage?. Am J Gastroenterol.

[5] Rotman SR, Bishop TF (2013). Proton pump inhibitor use in the U.S. ambulatory setting, 2002-2009. PLoS One.

[6] Ramirez E, Lei SH, Borobia AM, Pinana E, Fudio S, Munoz R (2010). Overuse of PPIs in patients at admission, during treatment, and at discharge in a tertiary Spanish hospital. Curr Clin Pharmacol.

[7] Parente F, Cucino C, Gallus S, Bargiggia S, Greco S, Pastore L (2003). Hospital use of acid-suppressive medications and its fall-out on prescribing in general practice: a 1-month survey. Aliment Pharmacol Ther.

[8] Grant K, Al-Adhami N, Tordoff J, Livesey J, Barbezat G, Reith D (2006). Continuation of proton pump inhibitors from hospital to community. Pharm World Sci.

[9] Nardino RJ, Vender RJ, Herbert PN (2000). Overuse of acid-suppressive therapy in hospitalized patients. Am J Gastroenterol.

[10] Forgacs I, Loganayagam A (2008). Overprescribing proton pump inhibitors. BMJ.

[11] Abraham NS (2012). Proton pump inhibitors: potential adverse effects. Curr Opin Gastroenterol.

[12] LUO HL, XIAO SL, WEI QM (2015). Investigation and analysis of the use of proton pump inhibitors in inpatients. Chinese J Drug Appl Monitor.

[13] XU ZL, LI YC, XIANG P (2014). Investigate the use of PPI to stress ulcer prophylaxis in 1745 cases of surgical patients receiving aseptic-contaminative and contaminative incision operation. Chongqing. Medicine.

[14] TANG JC (2017). Investigation and analysis of clinical application of proton pump inhibitors in surgical patients of our hospital. China Med Herald.

[15] Eom CS, Lee SS (2011). Risk of fracture and pneumonia from acid suppressive drugs. World J Methodol.

[16] Ho SW, Hsieh MJ, Yang SF, Yeh YT, Wang YH, Yeh CB (2015). Risk of stroke-associated pneumonia with acid-suppressive drugs: a population-based cohort study. Medicine (Baltimore).

[17] Chandra S, Agarwal D, Surana A (2013). Risk of Clostridium difficile infection with acid-suppressive therapy; pitfalls in accurate estimation. Am J Gastroenterol.

[18] Niu Q, Wang Z, Zhang Y, Wang J, Zhang P, Wang C, et al. Combination use of Clopidogrel and proton pump inhibitors increases major adverse cardiovascular events in patients with coronary artery disease: a meta-analysis. J Cardiovasc Pharmacol Ther. 2016; 10.1177/1074248416663647. 10.1177/1074248416663647 27512080

[19] Zhang HX, Li X, Huo HQ, Liang P, Zhang JP, Ge WH (2014). Pharmacist interventions for prophylactic antibiotic use in urological inpatients undergoing clean or clean-contaminated operations in a Chinese hospital. PLoS One.

[20] Liu HH, Liao GP (2016). The analysis of proton pump inhibitor use after clinical pharmacists intervention. China J Pharmaceut Econ.

[21] Ye ZK, Liu Y, Cui XL, Liu LH (2016). Critical appraisal of the quality of clinical practice guidelines for stress ulcer prophylaxis. PLoS One.

[22] Luo H, Fan Q, Xiao S, Chen K (2017). Impact of clinical pharmacist interventions on inappropriate prophylactic acid suppressant use in hepatobiliary surgical patients undergoing elective operations. PLoS One.

[23] Luo HLYY, Wang SR, Xiao SL, Liu D (2012). The analysis of prophylactic antibiotic use in perioperative period before and after antibiotic special campaign. Chongqing Med.

[24] Bez C, Perrottet N, Zingg T, Leung Ki EL, Demartines N, Pannatier A (2013). Stress ulcer prophylaxis in non-critically ill patients: a prospective evaluation of current practice in a general surgery department. J Eval Clin Pract.

[25] Hwang KO, Kolarov S, Cheng L, Griffith RA (2007). Stress ulcer prophylaxis for non-critically ill patients on a teaching service. J Eval Clin Pract.

[26] Hussain S, Stefan M, Visintainer P, Rothberg M (2010). Why do physicians prescribe stress ulcer prophylaxis to general medicine patients?. South Med J.

[27] Koczka CP, Geraldino-Pardilla LB, Goodman AJ (2013). Physicians’ opinions of stress ulcer prophylaxis: survey results from a large urban medical center. Dig Dis Sci.

[28] Ahn JS, Eom CS, Jeon CY, Park SM (2013). Acid suppressive drugs and gastric cancer: a meta-analysis of observational studies. World J Gastroenterol.

[29] ASHP Therapeutic Guidelines on Stress Ulcer Prophylaxis (1999). ASHP commission on therapeutics and approved by the ASHP Board of Directors on November 14, 1998. Am J Health Syst Pharm.

[30] Frelinger AL, Lee RD, Mulford DJ, Wu J, Nudurupati S, Nigam A (2012). A randomized, 2-period, crossover design study to assess the effects of dexlansoprazole, lansoprazole, esomeprazole, and omeprazole on the steady-state pharmacokinetics and pharmacodynamics of clopidogrel in healthy volunteers. J Am Coll Cardiol.

[31] Hulot JS, Collet JP, Silvain J, Pena A, Bellemain-Appaix A, Barthelemy O (2010). Cardiovascular risk in clopidogrel-treated patients according to cytochrome P450 2C19*2 loss-of-function allele or proton pump inhibitor coadministration. a systematic meta-analysis J Am Coll Cardiol.

[32] Zeng W, Finlayson AE, Shankar S, de Bruyn W, Godman B (2015). Prescribing efficiency of proton pump inhibitors in China: influence and future directions. BMC Health Serv Res.

[33] Tang Y, Zhang X, Yang C, Yang L, Wang H, Zhang X (2013). Application of propensity scores to estimate the association between government subsidy and injection use in primary health care institutions in China. BMC Health Serv Res.

[34] Armstrong D (2005). Intravenous proton pump inhibitor therapy: a rationale for use. Rev Gastroenterol Disord.

